# Effect of Native and Acetylated Dietary Resistant Starches on Intestinal Fermentative Capacity of Normal and Stunted Children in Southern India

**DOI:** 10.3390/ijerph16203922

**Published:** 2019-10-15

**Authors:** Ramadass Balamurugan, Srinivasan Pugazhendhi, Gowri M. Balachander, Tamilselvan Dharmalingam, Elissa K Mortimer, Geetha L. Gopalsamy, Richard J. Woodman, Rosie Meng, David H. Alpers, Mark Manary, Henry J. Binder, Ian L. Brown, Graeme P. Young, Balakrishnan S. Ramakrishna

**Affiliations:** 1 Wellcome Research Unit (Biochemistry), Christian Medical College, Vellore, Tamil Nadu 632004, India; balaramadass1@gmail.com (R.B.); pugazhs@gmail.com (S.P.); gowribalachandar@gmail.com (G.M.B.); dtamil_selvan8@yahoo.co.in (T.D.); 2 College of Medicine and Public Health, Flinders University of South Australia, Bedford Park 5045, South Australia, Australia; elissa.mortimer@flinders.edu.au (E.K.M.); gopa0006@gmail.com (G.L.G.); richard.woodman@flinders.edu.au (R.J.W.); Rosie.Meng@sa.gov.au (R.M.); graeme.young@flinders.edu.au (G.P.Y.); 3 Washington University School of Medicine, Saint Louis, MO 63110, USA; DAlpers@DOM.wustl.edu (D.H.A.); Manary@kids.wustl.edu (M.M.); 4 Yale University School of Medicine, New Haven, CT 06510, USA; Henry.binder@yale.edu; 5 Australian Cancer Research Foundation, Sydney 2000, Australia; mrbmrsb1@gmail.com; 6 Department of Gastroenterology, Christian Medical College, Vellore, Tamil Nadu 632004, India

**Keywords:** gut microbiota, colonic microbiota, human health, dysbiosis, therapeutic strategies, prebiotic, resistant starch

## Abstract

The health benefits of dietary amylase resistant starch (RS) arise from intestinal microbial fermentation and generation of short chain fatty acids (SCFA). We compared the intestinal fermentative capability of stunted and nonstunted (‘healthy’) children in southern India using two types of RS: high amylose maize starch (HAMS) and acetylated HAMS (HAMSA). Twenty children (10 stunted and 10 healthy) aged 2 to 5 years were fed biscuits containing HAMS (10 g/day) for two weeks followed by a 2-week washout and then HAMSA biscuits (10 g/day) for 2 weeks. Fecal samples were collected at 3-4 day intervals and pH and SCFA analyzed. At entry, stunted children had lower SCFA concentrations compared to healthy children. Both types of RS led to a significant decrease in fecal pH and increase in fecal acetate and propionate in both healthy and stunted children. However, while HAMS increased fecal butyrate in both groups of children, HAMSA increased butyrate in healthy but not stunted children. Furthermore, healthy children showed a significantly greater increase than stunted children in both acetate and butyrate when fed either RS. No adverse effects were reported with either RS. Stunted children have impaired capacity to ferment certain types of RS which has implications for choice of RS in formulations aimed at improving microbial function in stunted children.

## 1. Introduction

Normal growth and development in infancy and childhood are intimately linked to a functional intestine and absorptive system. Frequent enteric infections are important in the pathogenesis of growth-faltering and its consequences in children in low- and middle-income countries [1]. Alterations in the gut microbiome may arise from a variety of causes, including frequent enteric infections, and probably contribute to intestinal dysfunction in affected children [1,2]. 

One of the major functions of the colonic microbiota in health is to ferment dietary carbohydrate that has escaped digestion in the small intestine. It may be presumed that the colonic microbiota in children with stunting will be efficient in salvaging the unabsorbed carbohydrate through fermentation to short chain fatty acids (SCFA). However, this has never been tested. One of the major dietary carbohydrates is starch, which has components that can be digested by amylase in the small intestine (digestible starch) and also components that are resistant to amylase digestion in the small intestine (resistant starch, RS) [3]. The health benefits of dietary RS may derive from microbial fermentation and subsequent changes in colonic luminal pH and SCFA concentration [4,5]. RS is a preferred substrate for certain bacteria including bifidobacteria, being readily fermented by these bacteria into SCFA in the human large intestine [6,7]. Because of its broad effects on the composition of the gut luminal microbiota, RS is considered to be a prebiotic [8,9]. It is unclear if children from low-middle income countries ferment RS in the same way as healthy adults.

RS is categorized into five types (RS1-RS5) [10]. In RS1 the starch is physically inaccessible to digestion due to intact cell walls, e.g., partially milled grains; in RS2 the native starch granule structure protects it from digestion, e.g., high amylose maize starch (HAMS); in RS3 the starch is retrograded and thus inaccessible to amylase, e.g., cooked then cooled rice; in RS4 the starch is chemically modified to prevent accessibility to amylase, e.g., an acetylated starch as is used in the food industry; RS5 refers to amylose-lipid complexes [10]. Of these five types, RS2 and RS4 are the most attractive types of RS to be considered in dietary interventions as their content in food can be clearly defined and reproducibly maintained [11]. For these reasons we used HAMS (RS2) and acetylated HAMS (HAMSA) (RS4) in the present study.

HAMS is a source of RS that has received extensive interest in the medical literature regarding its health benefits [12,13,14]. HAMS is readily fermented by both adults and children, resulting in major changes in luminal pH and SCFA concentrations [14]. SCFA production reflects a healthy microbiota and SCFAs themselves have potential health benefits through their effects on epithelial metabolism, structure and mucosal integrity, immune function, cancer prevention, and fluid and electrolyte transport [5,9,13]. 

In terms of clinical application, the most obvious benefit has been the demonstration that RS consumption during acute diarrhea facilitates colonic salvage of fluid and electrolytes in adults and children when included in oral rehydration solutions as HAMS [12,13,14]. Based on animal studies in particular, the nature of the RS and the ecology of the microbes in the colon seem likely to influence both the capacity of an individual to ferment the starch substrate and the profile of the SCFA produced [4,15,16,17]. Esterified starches have been used to both limit small bowel digestion and to enhance delivery of SCFA to the colon. The SCFA are linked to the base starch with ester linkages and are rapidly released in the colon by microbial esterases that are abundantly present [18]. In addition to the release of SCFA from the esterified starches, the base starch can also be fermented by the colonic bacteria. Acetylated starch has been shown to potentiate the intestinal protective effects of bifidobacteria [19]. Stunted children are reported to have a dysbiosis or imbalance in the fecal microbiota [20]. We hypothesized that children living in an environment with suboptimal sanitation infrastructure in conjunction with microbial contamination of food and water sources would tolerate RS food supplements and that they would ferment the RS to SCFA with consequent reduction in fecal pH and increase in fecal SCFA concentration. In this study, we thus compared a naturally occurring RS with a modified (acetylated) RS in a sequential manner, and also evaluated differences between normally growing and stunted children.

## 2. Materials and Methods 

### 2.1. Design

A nonrandomized sequential feeding study was undertaken in March and April 2012 to determine whether short-term feeding of an RS, modified fecal parameters indicative of microbial fermentation. The trial was registered with the Australian New Zealand Clinical Trials Registry with the registration number ACTRN12618000182291. Primary outcome measures were pH and SCFA concentration of stool samples. Two RS sources were chosen because they are commercially available and widely used in the food industry: The first was HAMS (Hi-Maize, Ingredion Inc, Westchester, IL, USA) a RS2 obtained from a natural cultivar of maize and the second a RS4, which was a chemically acylated HAMS (HAMSA) with 2.5% acetylation (Crisp Film, Ingredion Inc, Westchester, IL, USA). HAMS contained approximately 42% RS while HAMSA contained closer to 50% RS [21,22]. The remainder was slowly digested starch [21,22]. Participants were stratified into either healthy or stunted children. There was a 2-week washout period between the two RS interventions to allow the colonic luminal environment and microflora to stabilize [23]. The predetermined order of administration was HAMS-washout-HAMSA. We based this on the reasoning that both supplements contained HAMS as a base starch, thus HAMSA should logically follow HAMS administration after a washout period. 

### 2.2. Study Protocol

**Figure 1:**
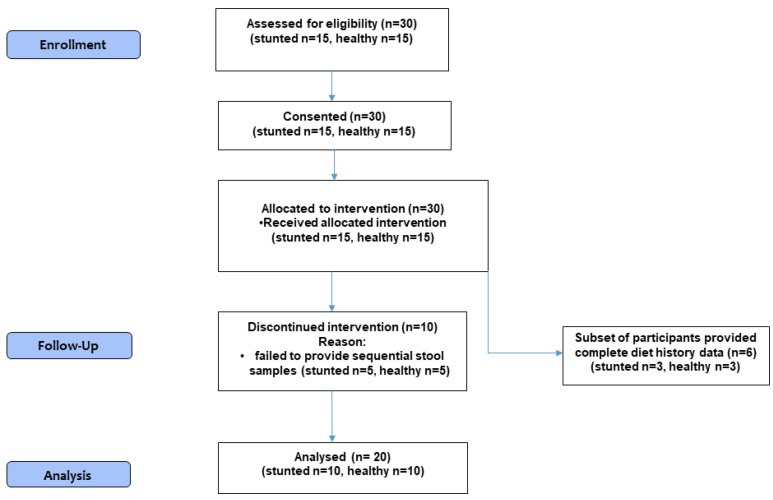
Participant flow diagram.

Participant recruitment and enrolment using community-based sampling in a village within 30 km of Vellore, Tamil Nadu, south India, began on 5 March 2012 and concluded on 21 March 2012 (Figure 1). The last date for participant follow-up and data collection was 14 May 2012. Focus group discussions with Anganwadi (rural child care centers) and community leaders was first undertaken. Subsequently, a list of potential participants was made and the first 30 children who met the eligibility requirements were recruited. Thirty children aged 2 to 5 years were enrolled. The 30 children were divided into 15 without stunting and 15 with stunting based on Height-for-Age according to WHO Child Growth Standards [24]. 

Inclusion criteria stated that children were to be aged 2–5 years. Exclusion criteria were stipulated as: Any chronic illness, including Grade IV malnutrition, with or without symptoms of peripheral edema, or a Height-for-Age z-score (HAZ) below minus 3 standard deviations of the WHO reference value. 

Participants were provided with biscuits containing 10 g/day HAMS, equivalent to approximately 4.2 g/day of RS, which they consumed every day for two weeks, in addition to their usual diet, under supervision by the study team. Resistant starch content was measured in representative samples of HAMS and HAMSA biscuits using the Megazyme Resistant Starch Assay kit (K-RSTAR 08/11, Megazyme International, Wicklow, Ireland). Average values were obtained from analysis of 6 biscuits in duplicate, and mean daily consumption of RS was calculated for each participant based on number of biscuits eaten. Participants began consumption of the biscuits on day 1 and continued for a two-week period. After a two-week washout period during which participants consumed their habitual diet, they then consumed 10 g/day HAMSA (equivalent to approximately 5 g of RS/day) as biscuits for two weeks in addition to their usual diet. The study design was open-label. Originally, the study protocol indicated that the order of HAMS/HAMSA consumption would be randomized in a crossover design. However, because the base starch was the same in HAMS and HAMSA and the preliminary feasibility testing indicated that outcome parameters (pH and SCFA) returned to baseline at the end of the washout period, a sequential feeding study design was used. Stool samples were collected from the participant’s home in the morning on days 0, 3, 7, 10, 15, 18, 22, 25, 29, 32, 36, 39, and 44, and transported on ice to the laboratory where they were aliquoted and stored at -80 °C until analysis.

### 2.3. Target Sample Size and Rationale

In this study, we measured fecal pH before and after starch (i.e., paired observations) in each participant. A priori, we assumed that fecal pH would be normally distributed with a standard deviation of 2, and to be able to detect a mean pH difference of 2 between the paired samples with a type I error of 0.05 and a power of 80%, the sample size was calculated as 10 participants.

### 2.4. Dietary Composition Analysis

Collection of all data for the study was undertaken at the participant’s home. Children’s height and weight were recorded at study enrolment. Habitual dietary intake was assessed in subsets of 3 representative children from each of the healthy and stunted groups. Dietary intakes of food groups were calculated from 24-hour dietary recall on four different days of the study and a food frequency questionnaire of commonly used foods consumed over the previous 3 months. These tools were administered by the study dietitian with the primary caregivers of study participants in their local language using standard local measures to determine quantity. Dietary intake was then analyzed by the study dietitian for nutrient content using reference values for Indian foods [25]. Given that study participants were preschool-aged children, the majority of their time was spent with their primary caregivers throughout the study period. The caregivers could, therefore, confidently report the entire dietary intake of participants accurately. Dietary composition results are provided in Tables S1 and S2.

### 2.5. HAMS and HAMSA Consumption

Biscuits incorporating either one of the starches were prepared in a commercial facility in Vellore. Each day, a study staff member who resided locally, delivered biscuits containing 10 g of the appropriate starch to the participant’s home. Participants consumed biscuits immediately under direct observation by study personnel. Any leftover biscuits were weighed and daily HAMS or HAMSA intake was estimated. 

### 2.6. Measurement of pH and SCFA

Fecal pH was measured in a Beckman pH meter using an emulsion of feces in normal saline [26]. Fecal SCFA were extracted by vacuum distillation under ice. Briefly, 1 mL of internal standard mixture (equimolar mix of 0.5 mM/L of isobutyric and isovaleric acids, analytical grade) was added to 1 g stool sample, vortexed while cold, and 400 µL of this sample with 160 µL of 20% of orthophosphoric acid was subjected to vacuum distillation under ice (ethanol at −800 °C) through successive bulbs. SCFA concentrations were measured by gas chromatography in a Shimadzu GC-MS QP 2010 Plus (Shimadzu Corporation, Kyoto, Japan) using a Stabilwax-DA (60 m, 0.25 mm ID, 0.5 µm df) column. One microliter of each sample was injected into the Gas Chromatograph (GC) using an AOC-20s auto injector. Oven temperature was 1000 °C with a hold time of 2 minutes, ramp at 250 °C per minute to 1800 °C and ramp at 100 °C/min to 2300 °C where it was held for 5 min. The total run time was 15.2 min. The carrier gas was helium at pressure of 228.1 kPa, total flow of 24.5 mL/min, column flow 1.77 mL/min, linear velocity 35.0 cm/sec, purge flow 5.0 mL/min, and split ratio 10. The parameters used in the Mass Spectrometer (MS) were an ion source temperature of 200 °C with scanning time of 7 to 11 min. SCFA were quantitated against the internal standard. Initial standardization was achieved by injecting individual SCFA (acetate, propionate, butyrate, and valerate) standards at concentrations of 0.25 mM, 0.5 mM, 1 mM, and 2 mM for calibration.

### 2.7. Ethics

This study was carried out in accordance with the Declaration of Helsinki of 1975, as revised in 2013, and received ethical approval from the Southern Adelaide Clinical Human Research Ethics Committee, South Australia (App Number: 079.12, date 01.03.2012) and the Institutional Review Board of Christian Medical College, Vellore, India (Approval No. 761 date 23.11.2011). Informed written consent for each participant was obtained from their parents/caregivers and recorded on the Participant Information Sheet and Consent Form as per the study protocol.

### 2.8. Statistical Analysis

All analyses were performed using STATA version 13.1 (StataCorp, TX, USA). Differences in participant characteristics between treatment groups were assessed using independent t-tests and chi-squared tests as appropriate. The effect of the two RS treatments on propionate, butyrate, acetate, and pH was compared with that during consumption of habitual diet. Differences between the healthy and stunted children were assessed using linear mixed effects models to account for the 13 separate measurements recorded on each child. The main model included fixed effect terms for each of the 4 periods (day 0, days 7-15 (HAMS), days 22-29 (washout) and days 36-45 (HAMSA)), group (healthy or stunted), and a categorical period and group interaction term. To increase precision, an additional adjustment for baseline periods using the dependent variable values on days 0 and day 29 (the last days of each baseline period) was included. Participant identity was included as a random effect. Both overall and separate group effects (healthy and stunted) were reported for the HAMS and HAMSA periods and for the HAMSA versus HAMS periods. Between-group differences (stunted versus healthy) for the HAMS and HAMSA periods were determined. In a supplementary day-by-day analysis to assess the effects of the 2 treatments on individual days, a model that included fixed effect terms for day (as a categorical variable), treatment (baseline/washout, HAMS, HAMSA), and group (healthy or stunted) was used. A two-sided type 1 error rate of 5 % was used for all statistical analyses.

## 3. Results

The families of 30 children (15 with normal HAZ (‘healthy’) and 15 stunted) initially consented for the study. However, five children each in both the healthy and stunted groups failed to provide sequential stool samples as per the protocol and thus withdrew from the study. A total of 20 children (10 healthy and 10 stunted) completed the study protocol, and were included in the per protocol analysis. 

Caregivers of study participants were questioned every day to determine if participating children had experienced abdominal pain, alteration of bowel habit, or other digestive symptoms which the caregiver attributed to the dietary supplement. When feasible, the study participants were similarly questioned. None of the children or their caregivers reported any such adverse effects during the study period. 

### 3.1. Participant Characteristics

Table 1 shows the characteristics of the 20 participants on day zero of the study and the amounts of HAMS/HAMSA consumed during the study. There were significant differences between healthy and stunted children in their HAZ scores, reflecting the distribution of nutritional status in the general population [20]. Fecal concentrations of each of the 3 SCFA were lower in stunted children. Biscuit consumption was the same for stunted and healthy children. Energy intakes were similar in both groups as assessed from 3 participants in each group (726 ± 190 kcal per day and 656 ± 121 kcal per day, respectively, in healthy and stunted children, mean ± SD) (*p* 0.29).

### 3.2. Fecal pH

**Figure 2:**
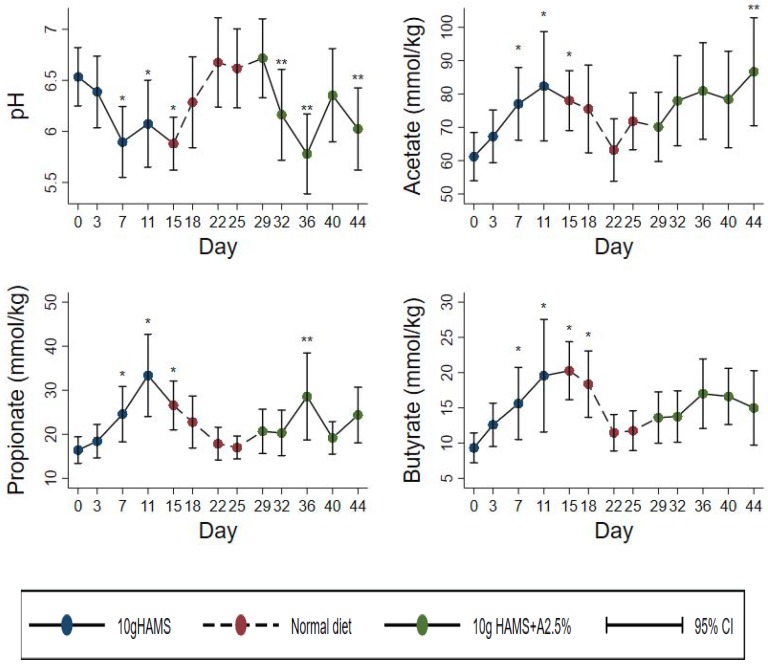
Fecal parameters of RS fermentation (pH and SCFA) in all participants over the study period (n = 20). Values are mean and 95% CI. * Significantly higher versus day 0: *p* < 0.05. ** Significantly higher versus day 29: *p* < 0.05.

RS effect overall: Fecal pH was significantly lower during both the HAMS (*p* < 0.01) and HAMSA (*p* < 0.05) supplementation periods (Table 2, Figure 2) compared to the basal period. However, there was no significant difference between HAMS and HAMSA (*p* = 0.41) supplementation. Fecal pH returned rapidly to baseline during the washout period. 

**Figure 3:**
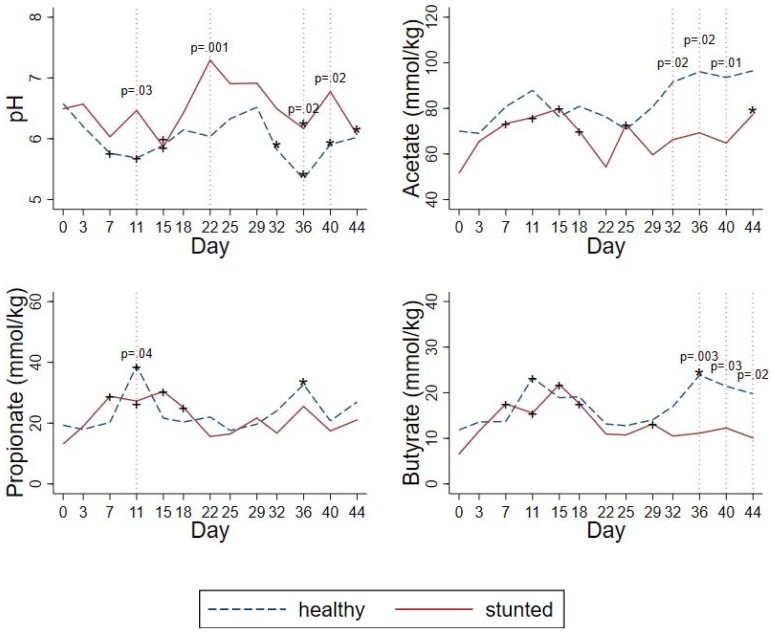
Baseline adjusted fecal pH and SCFA showing differences between healthy and stunted children (n = 20). Values are adjusted mean. + *p* < 0.05 versus day 0. * *p* < 0.05 versus day 29.

Comparison of healthy and stunted children: Both healthy and stunted children showed a reduction in fecal pH which was similar for both the HAMS and HAMSA supplementation periods (Table 3 and Figure 2). In the day-by-day analysis, healthy children had a lower baseline-adjusted pH than stunted children at day 11 (on HAMS, p=0.03), at day 22 (washout, *p* = 0.001), day 36 (*p* = 0.02) (on HAMSA), and day 40 (*p* = 0.02) (on HAMSA) (Figure 3).

### 3.3. Fecal Acetate

RS effect overall: Fecal acetate concentration increased significantly (*p* < 0.01) in both HAMS and HAMSA periods, and returned to normal levels during the washout period (Table 2, Figure 2). There was no significant difference in the difference between HAMS and HAMSA periods (*p* = 0.52). 

Comparison of healthy and stunted children: Fecal acetate concentrations rose in both groups but to a different degree between the RS types as shown in Figure 3 and Table 2 and Table 3. There was no difference between healthy and stunted children in the rise in acetate concentrations during the HAMS intervention period (Table 3, *p* = 0.41). However, the rise in acetate was substantially and significantly higher in the healthy compared to the stunted group during the HAMSA intervention (Table 3, *p* < 0.001). In the day-by-day analysis, healthy children had higher acetate than stunted children at day 32, 36, and 40 during the HAMSA intervention (Figure 3).

### 3.4. Fecal Propionate

RS effect overall: Propionate concentrations were significantly increased in the HAMS period (*p* < 0.001) and to a lesser degree in the HAMSA period (*p* = 0.03) (Table 2, Figure 2), although the difference between HAMS and HAMSA did not quite reach significance (Table 2, *p* = 0.09). 

Comparison of healthy and stunted children: There were no differences between healthy and stunted children in fecal propionate concentrations with either HAMS (*p* = 0.65) or HAMSA (*p* = 0.17) (Table 3 and Figure 3). In the day-by-day analysis, healthy children had higher fecal propionate than stunted children only at day 11 in the HAMS period (Figure 3).

### 3.5. Fecal Butyrate

RS effect overall: Butyrate was increased significantly in the HAMS period (*p* < 0.001), and also in the HAMSA period (*p* = 0.025) (Table 2, Figure 2), although the difference between HAMS and HAMSA was not significant (*p* = 0.24). 

Comparison of healthy and stunted children: In healthy children, fecal butyrate increased significantly during both HAMS (*p* < 0.05) and HAMSA (*p* < 0.01) feeding (Table 2). However, among stunted children, butyrate increased only during HAMS (*p* < 0.01) but not during HAMSA feeding (Table 2). However, the rise in butyrate was significantly higher in the healthy compared to the stunted group during the HAMSA intervention (Table 3, *p* < 0.001). In the day-by-day analysis, healthy children had higher butyrate than stunted children at day 32, 36, and 40 in the HAMSA period (Figure 3).

## 4. Discussion

The major finding of this study was that the two sources of RS, HAMS and HAMSA, were fermented in the bowel of preschool child participants, with substrate-responsive fecal SCFA levels being lower in stunted compared to healthy children. This could reflect reduced SCFA generation from these substrates in stunted children, although increased absorption of the generated SCFA is also a possibility. Taken together they reflect differences in colonic physiology between healthy and stunted children. 

RS escapes digestion in the small bowel and reaches the colon where it is fermented by the colonic bacteria to a variety of SCFA [10,11]. Bacterial fermentation of RS is a complex process that likely involves a consortium of bacterial species. In contrast to HAMS, which releases SCFA solely as a result of microbial fermentation in the colon, HAMSA has the capacity to release acetate as a result of hydrolysis of the ester bond and cleave acetate from the starch by bacterial enzymes [18]. Acetate released from HAMSA will not remain solely as acetate but will, through bacterial metabolism, be converted into other SCFA such as propionate or butyrate [27]. 

Both the RS formulations used in this study altered bowel fermentation, as evidenced by the increase in fecal SCFA (acetate, propionate, and butyrate) and decrease in fecal pH. The pH changes in response to feeding RS were established quickly and reverted to baseline during the washout period. This suggested a dynamic responsiveness in which pulses of RS might be more effective than sustained long-term administration. This has implications for future implementation of resistant starch interventions at the population or target group level where ensuring daily compliance with an intervention might be constrained by contextual factors, particularly in the low-middle income country context. All three SCFA were noted to increase with both types of RS in healthy children, with acetate being significantly higher when HAMSA was consumed compared to HAMS (*p* = 0.04, Table 2). This would be expected given that acetate is linked onto the starch backbone in HAMSA. It is not known whether the acetyl esters linked to starch impair the fermentability of the native starch in the event that microbial esterases are lacking in the colon. If so, a deficiency in microbial esterases may also potentially be responsible for lower fecal acetate levels in stunted children fed acetylated HAMS.

Differences in the fermentation parameters were noted between healthy and stunted children. At entry, all stunted children had lower fecal SCFA concentrations compared to healthy children. Both types of RS led to a decrease in fecal pH. Both RS formulations increased fecal acetate but healthy children had a significantly greater increase in acetate compared to stunted children after HAMSA feeding (Table 3). Similarly, both RS increased fecal butyrate in healthy children while HAMS, but not HAMSA, increased fecal butyrate in stunted children. 

Energy and carbohydrate malabsorption from the small intestine can occur when Environmental Enteropathy (EE) is present [28], a condition associated with stunting which results from repeated exposure to environmental pathogens [29]. A high prevalence of stunting in low-middle income countries has been attributed to dysfunction of the small intestine [29,30]. Prebiotic interventions with RS have been mooted as a way to improve gut health [31,32]. In a limited study in children, four children (three adolescent, one prepubertal) consumed a HAMS-RS2-enriched yogurt for four weeks to test its fermentability. The adolescents had reduced stool pH and increased stool SCFA including acetate and butyrate [33]. Our investigation represents the first assessment of fecal SCFA concentrations after oral administration of two types of RS in substantially younger children who are stunted. Fecal SCFA concentration has been used extensively as a measure of carbohydrate fermentation in both human and animal studies [34,35]. The fermentation of carbohydrate to SCFA is a cooperative activity requiring microbes with different enzymatic and metabolic capabilities. Reduced SCFA levels in the stunted participants likely reflects reduced production from the substrate, although increased absorption of SCFA from the colon (an energy-conserving function in stunted children) cannot be excluded. Whatever the cause, lower fecal levels of SCFA in response to an RS in stunted children reflect altered colonic physiology in these children. It is possible that there is an alteration of gut microbial mass or of relative composition in these children. There is limited information on the composition of the gut microbiota of stunted children, with changes not being consistent across studies [36,37].

Additional issues flagged here may limit interpretation of the current study. Data on diarrheal disease were collected only for the period immediately prior to and during the study, and none of the children experienced diarrhea during this period. Unfortunately we did not collect information regarding frequency of diarrheal illness in the first year of life, which is a factor reportedly associated with stunting [38,39,40]. A second limitation is that diet data were obtained in only 6 (3 normal and 3 stunted) participants overall, albeit at four different time points in each participant. As participants were drawn from similar backgrounds it was considered reasonable to extrapolate this to the entire group. The lack of a randomized crossover design might be a limitation of the study. We deliberately chose not to have a randomized crossover design because the base starch was the same in HAMSA and HAMS and parameters had returned to baseline at the end of the washout period. However, it is possible that the first intervention already led to a longer-term change in microbial composition and fermentative capacity that carried through to the period of the second intervention. The high dropout rate may also be a limitation. However, it is noteworthy that the number of participants who completed the study did actually meet the intended sample size.

## 5. Conclusions

In conclusion, both the RS2 and RS4 provide substrate for fermentation by intestinal bacteria and the study provides clear evidence that feeding of these starches resulted in increased fermentation in preschool children in southern India. However, stunted children failed to generate the same high levels of acetate and butyrate during HAMSA supplementation as did healthy children, pointing to altered colonic physiology in these children. SCFA, particularly butyrate, are important to colonic health, especially for the colonic epithelium [27]. These findings have implications for consideration of supplementation of RS in efforts to ameliorate the physiological effects of stunting in children residing in low- and middle-income countries. 

## Figures and Tables

**Table 1:** Participant characteristics and high amylose maize starch (HAMS) and acetylated HAMS (HAMSA) consumption.

	Healthy (n = 10)	Stunted (n = 10)	*p*-Value ^1^
Age in month, mean (SD)	40.0 (7.6)	41.9 (13.3)	0.93
Male (%)	40%	60%	0.37
HAMS intake in g/day, mean (SD)	8.78 (1.88)	9.44 (0.86)	0.33
HAMSA intake in g/day, mean (SD)	8.88 (1.81)	10.14 (1.65)	0.09
Fecal parameters			
Day 0 pH	6.6 (0.7)	6.5 (0.7)	0.78
Day 0 acetate (mmol/Kg)	70.0 (15.9)	51.4 (9.8)	0.007
Day 0 propionate (mmol/Kg)	19.3 (6.5)	13.2 (5.7)	0.02
Day 0 butyrate (mmol/Kg)	11.8 (5.1)	6.5 (2.0)	0.004

^1^ Using t-test or chi-squared test as appropriate. ^2^ From 6 randomly selected participants (n = 3 healthy & n = 3 stunted).

**Table 2:** Parameters of fecal fermentation (pH and short chain fatty acids (SCFA) concentrations, mmol/Kg feces) according to growth status at baseline and at the end of specific resistant starch (RS) interventions, together with differences between HAMS and HAMSA.

	Day 0(Commencement)	Day 7–15 (2nd Week of HAMS)	Days 22–29 (2nd Week of Washout)	Days 36–44 (2nd Week of HAMSA)	Difference ^1^ (HAMSA versus HAMS)	*p*-Value ^1^
	Mean (SD)	Mean (SD)	Mean (SD)	Mean (SD)	Mean (SE)	
Overall (n = 20)						
Fecal pH	6.5 (0.7)	6.0 (0.8)^b^	6.7 (0.9)	6.1 (0.9)^a^	0.11 (0.14)	0.41
Acetate	61.2 (16.1)	79.1 (27.5)^b^	68.5 (21.2)	81.9 (32.5)^b^	2.84 (4.46)	0.52
Propionate	16.4 (6.7)	28.1 (16.4)^c^	18.5 (8.8)	23.9 (15.5)^a^	−3.98 (2.34)	0.09
Butyrate	9.3 (4.7)	18.4 (13.3)^c^	12.3 (6.9)	16.2 (10.2)^a^	−1.97 (1.70)	0.24
Healthy (n = 10)						
Fecal pH	6.6 (0.7)	5.8 (0.7)^b^	6.3 (0.8)	5.8 (0.8)^b^	0.11 (0.20)	0.58
Acetate	70.0 (15.9)	81.7 (30.7)	75.3 (23.6)	95.1 (32.4)^c^	13.3 (6.4)	0.04
Propionate	19.3 (6.5)	27.3 (18.5)	19.2 (8.5)	26.7 (16.4)^a^	−0.54 (3.28)	0.87
Butyrate	11.8 (5.1)	18.5 (15.4)^a^	13.0 (6.7)	21.8 (11.6)^b^	2.91 (2.37)	0.22
Stunted (n = 10)						
Fecal pH	6.5 (0.7)	6.1 (0.9)	7.0 (0.8)^b^	6.3 (1.0)^c^	0.12 (0.20)	0.54
Acetate	51.4 (9.8)	76.4 (24.3)^b^	62.2 (16.8)	70.1 (28.2)	−6.24 (6.21)	0.32
Propionate	13.2 (5.7)	28.9 (14.1)^b^	17.9 (9.2)^a^	21.5 (14.5)	−7.51 (3.35)	0.025
Butyrate	6.5 (2.0)	18.3 (10.9)^b^	11.6 (7.0)^a^	11.2 (5.1)	−6.82 (2.43)	0.005

^1^ Baseline adjusted mean differences (SE) and *p*-value using mixed models comparing HAMS with HAMSA. Comparisons in levels between specific time points, when significant, are shown as follows: a *p* < 0.05; *bp* < 0.01; *cp* < 0.001 (when comparing days 7–15 with day 0, days 22–29 with day 0, and days 36–44 with day 29.).

**Table 3:** Differences in fecal parameters of fermentation between treatments in healthy and stunted children.

	Healthy versus Stunted (Mean Difference ± SE)		Healthy versus Stunted (Mean Difference ± SE)	
Parameter	Day 7–15 (HAMS Period)	*p*-Value ^1^	Days 36–44 (HAMSA Period)	*p*-Value ^1^
Fecal pH	−0.24 ± 0.20	0.23	−0.25 ± 0.20	0.21
Acetate	5.42 ± 6.58	0.41	24.95 ± 6.75	<0.001
Propionate	−1.69 ± 3.77	0.65	5.27 ± 3.85	0.17
Butyrate	0.52 ± 2.80	0.85	10.25 ± 2.86)	<0.001

Effects were calculated using a linear mixed model with fixed effect terms for period, baseline values, and group (healthy (n = 10) versus stunted (n = 10)). There is ^1^ *p*-value for difference between stunted and normal growth children.

## Supplementary Materials

The following are available online at https://www.mdpi.com/1660-4601/16/20/3922/s1, Table S1: Dietary composition analysis of 3 healthy participants. Table S2: Dietary composition analysis of 3 stunted participants. Table S3: TREND Statement Checklist. Table S4: Participant biodata. Table S5: Nutrient composition-study biscuits.
