# Supplementary material for: Effect of Native and Acetylated Dietary Resistant Starches on Intestinal Fermentative Capacity of Normal and Stunted Children in Southern India
Source: Int J Environ Res Public Health. 2019 Oct 15;16(20):3922. doi: 10.3390/ijerph16203922 (PMC6843365; doi:10.3390/ijerph16203922)
Supplement: Supplementary file 1 [file ijerph-16-03922-s001.zip › IJERPH_Rev1_Supp_materials/Table S1.docx]

| **Participant code** | | **Dietary data collected date** | **Sample No** | **Energy (kcals)** | **Protein (gms)** | **Carbohydrate (gms)** | **Fat (gms)** | **Iron (mg)** | **Calcium (mg)** | **Fibre (gm)** | **Phosphorus (mg)** | **Magnesium (mg)** | **Sodium (mg)** | **Potassium (mg)** | **Copper (mg)** |
| --- | --- | --- | --- | --- | --- | --- | --- | --- | --- | --- | --- | --- | --- | --- | --- |
| 1 | HK - 20 | 22.03.12 | 1 | 454.2 | 13.1 | 74.3 | 11.7 | 1.0 | 272.8 | 0.4 | 304.2 | 88.5 | 153.0 | 467.7 | 0.2 |
|  |  | 10.04.12 | 5 | 785.4 | 24.7 | 144.7 | 11.8 | 3.8 | 93.4 | 0.8 | 458.2 | 211.7 | 13.4 | 342.9 | 0.4 |
|  |  | 24.04.12 | 9 | 723.2 | 18.5 | 150.4 | 5.2 | 2.7 | 65.9 | 0.9 | 358.2 | 211.7 | 16.2 | 404.7 | 0.4 |
|  |  | 7.05.12 | 13 | 648.0 | 19.9 | 120.2 | 10.3 | 5.5 | 181.9 | 1.9 | 498.3 | 185.1 | 91.9 | 506.7 | 0.5 |
|  |  |  |  |  |  |  |  |  |  |  |  |  |  |  |  |
| 2 | HK - 38 | 22.03.12 | 1 | 796.5 | 23.3 | 128.3 | 21.2 | 1.5 | 519.0 | 1.1 | 574.6 | 142.1 | 299.2 | 832.9 | 0.3 |
|  |  | 8.04.12 | 5 | 1079.5 | 39.6 | 167.6 | 27.9 | 2.6 | 546.6 | 0.4 | 789.8 | 168.1 | 293.7 | 587.3 | 0.0 |
|  |  | 22.04.12 | 9 | 846.3 | 23.7 | 140.5 | 21.2 | 3.7 | 613.7 | 1.6 | 604.4 | 159.6 | 307.4 | 926.5 | 0.4 |
|  |  | 7.05.12 | 13 | 895.9 | 24.8 | 153.9 | 20.4 | 2.6 | 522.4 | 1.0 | 633.5 | 164.6 | 302.6 | 801.2 | 0.3 |
|  |  |  |  |  |  |  |  |  |  |  |  |  |  |  |  |
| 3 | HK - 22 | 23.03.12 | 1 | 429.3 | 8.6 | 77.6 | 8.5 | 1.0 | 141.9 | 0.8 | 204.4 | 99.9 | 74.7 | 167.3 | 0.0 |
|  |  | 9.04.12 | 5 | 521.3 | 14.5 | 79.8 | 14.4 | 2.3 | 274.4 | 1.4 | 363.8 | 85.6 | 153.2 | 367.6 | 0.1 |
|  |  | 23.04.12 | 9 | 832.1 | 21.1 | 148.0 | 17.4 | 4.6 | 469.8 | 0.5 | 516.1 | 147.8 | 263.2 | 546.3 | 0.2 |
|  |  | 7.05.12 | 13 | 701.3 | 20.8 | 116.1 | 17.2 | 5.4 | 513.2 | 0.9 | 503.0 | 124.5 | 287.7 | 720.4 | 0.3 |
|  | Mean |  |  | 726.1 |  |  |  |  |  |  |  |  |  |  |  |

| **Participant code** | | **Manganese (mg)** | **Zinc (mg)** | **Sulphur (mg)** | **Carotene (mcg)** | **Thiamine (mg)** | **Riboflavin (mg)** | **Niacin (mg)** | **Folic acid (mcg)** | **Vitamin C (mg)** | **Choline (mg)** | **Arginine (mg)** | **Histidine (mg)** | **Lysine (mg)** | **Tryptophan (mg)** |
| --- | --- | --- | --- | --- | --- | --- | --- | --- | --- | --- | --- | --- | --- | --- | --- |
| 1 | HK - 20 | 0.5 | 1.0 | 229.7 | 179.2 | 0.3 | 0.4 | 2.4 | 44.0 | 7.8 | 31.2 | 768.5 | 390.5 | 883.4 | 156.3 |
|  |  | 1.6 | 3.0 | 97.8 | 302.0 | 0.5 | 0.4 | 6.9 | 100.1 | 3.8 | 66.1 | 2019.9 | 721.1 | 1370.6 | 281.3 |
|  |  | 1.6 | 3.1 | 69.5 | 98.0 | 0.5 | 0.2 | 7.1 | 61.0 | 8.1 | 91.1 | 1615.5 | 567.9 | 922.9 | 191.9 |
|  |  | 2.2 | 2.5 | 209.8 | 146.8 | 0.6 | 0.4 | 5.7 | 67.2 | 4.5 | 18.3 | 982.7 | 458.9 | 786.2 | 197.0 |
|  |  |  |  |  |  |  |  |  |  |  |  |  |  |  |  |
| 2 | HK - 38 | 1.0 | 1.7 | 445.3 | 326.5 | 0.5 | 0.9 | 5.1 | 77.7 | 16.6 | 48.3 | 1320.7 | 689.5 | 1541.3 | 284.5 |
|  |  | 1.2 | 2.2 | 458.6 | 487.1 | 0.6 | 1.1 | 6.2 | 96.0 | 11.8 | 0.0 | 2442.6 | 1066.2 | 2664.4 | 488.8 |
|  |  | 2.1 | 1.9 | 474.0 | 529.8 | 0.6 | 0.9 | 4.5 | 66.3 | 14.7 | 31.9 | 1281.5 | 678.5 | 1538.3 | 295.3 |
|  |  | 1.5 | 2.2 | 453.0 | 292.5 | 0.6 | 0.9 | 5.8 | 67.9 | 13.5 | 28.3 | 1383.0 | 697.2 | 1514.5 | 307.6 |
|  |  |  |  |  |  |  |  |  |  |  |  |  |  |  |  |
| 3 | HK - 22 | 0.6 | 1.1 | 101.1 | 118.1 | 0.2 | 0.2 | 3.0 | 20.4 | 5.8 | 0.0 | 607.4 | 234.3 | 430.5 | 101.6 |
|  |  | 0.5 | 0.8 | 216.4 | 354.3 | 0.3 | 0.4 | 2.5 | 47.5 | 4.3 | 26.0 | 620.8 | 317.3 | 713.6 | 135.7 |
|  |  | 1.1 | 2.4 | 312.0 | 306.2 | 0.6 | 0.9 | 6.8 | 94.4 | 20.9 | 12.9 | 1244.2 | 534.3 | 1137.1 | 241.9 |
|  |  | 0.8 | 2.3 | 344.7 | 358.2 | 0.6 | 1.1 | 6.5 | 112.6 | 34.8 | 48.1 | 1169.3 | 536.6 | 1232.7 | 237.2 |

| **Participant code** | | **Phenylalanine (mg)** | **Tyrosine (mg)** | **Methionine (mg)** | **Cystine (mg)** | **Threonine (mg)** | **Palmitic acid (gm)** | **Stearic acid (gm)** | **Arachidic acid (gm)** | **Oleic acid (gm)** | **Linoleic acid (gm)** | **Linolenic acid (gm)** | **EPA** | **DHA** | **Total PUFA (gm)** |
| --- | --- | --- | --- | --- | --- | --- | --- | --- | --- | --- | --- | --- | --- | --- | --- |
| 1 | HK - 20 | 726.5 | 521.6 | 306.8 | 136.6 | 542.5 | 0.4 | 0.1 | 0.1 | 1.4 | 0.9 | 0.0 | 0.0 | 0.0 | 0.9 |
|  |  | 1415.9 | 916.4 | 673.7 | 398.7 | 1071.6 | 1.6 | 0.5 | 0.2 | 3.6 | 1.9 | 0.0 | 0.0 | 0.0 | 2.1 |
|  |  | 1050.1 | 661.3 | 456.0 | 252.9 | 745.2 | 0.5 | 0.1 | 0.2 | 1.9 | 1.2 | 0.0 | 0.0 | 0.0 | 1.2 |
|  |  | 907.1 | 608.5 | 344.8 | 296.4 | 619.8 | 0.5 | 0.1 | 0.2 | 1.9 | 1.2 | 0.0 | 0.0 | 0.0 | 1.2 |
|  |  |  |  |  |  |  |  |  |  |  |  |  |  |  |  |
| 2 | HK - 38 | 1285.8 | 1012.7 | 592.7 | 239.6 | 999.8 | 0.2 | 0.1 | 0.0 | 2.3 | 1.2 | 0.0 | 0.0 | 0.0 | 2.4 |
|  |  | 1993.9 | 1689.3 | 1155.6 | 537.6 | 1775.5 | 3.1 | 1.1 | 0.2 | 6.8 | 3.8 | 0.1 | 0.0 | 0.0 | 4.2 |
|  |  | 1289.4 | 998.0 | 596.3 | 258.7 | 988.8 | 0.4 | 0.1 | 0.1 | 1.4 | 0.9 | 0.0 | 0.0 | 0.0 | 0.9 |
|  |  | 1320.4 | 1057.3 | 620.5 | 293.6 | 1034.1 | 0.4 | 0.1 | 0.1 | 1.4 | 0.9 | 0.0 | 0.0 | 0.0 | 0.9 |
|  |  |  |  |  |  |  |  |  |  |  |  |  |  |  |  |
| 3 | HK - 22 | 436.1 | 384.0 | 252.2 | 102.4 | 367.8 | 0.5 | 0.1 | 0.2 | 1.9 | 1.2 | 0.0 | 0.0 | 0.0 | 1.2 |
|  |  | 592.0 | 486.0 | 288.2 | 114.0 | 473.9 | 0.6 | 0.1 | 0.2 | 2.4 | 1.5 | 0.0 | 0.0 | 0.0 | 1.4 |
|  |  | 995.0 | 859.2 | 536.2 | 217.6 | 837.2 | 0.5 | 0.1 | 0.2 | 1.9 | 1.2 | 0.0 | 0.0 | 0.0 | 1.2 |
|  |  | 1004.8 | 797.5 | 474.0 | 204.5 | 809.6 | 0.5 | 0.1 | 0.2 | 1.9 | 1.2 | 0.0 | 0.0 | 0.0 | 1.2 |
