# Supplementary material for: Effect of Native and Acetylated Dietary Resistant Starches on Intestinal Fermentative Capacity of Normal and Stunted Children in Southern India
Source: Int J Environ Res Public Health. 2019 Oct 15;16(20):3922. doi: 10.3390/ijerph16203922 (PMC6843365; doi:10.3390/ijerph16203922)
Supplement: Supplementary file 1 [file ijerph-16-03922-s001.zip › IJERPH_Rev1_Supp_materials/Table S2.docx]

| **Participant code** | | **Dietary data collected date** | **Sample No** | **Energy (kcals)** | **Protein (gms)** | **Carbohydrate (gms)** | **Fat (gms)** | **Iron (mg)** | **Calcium (mg)** | **Fibre (gm)** | **Phosphorus (mg)** | **Magnesium (mg)** | **Sodium (mg)** | **Potassium (mg)** | **Copper (mg)** |
| --- | --- | --- | --- | --- | --- | --- | --- | --- | --- | --- | --- | --- | --- | --- | --- |
| 1 | HK 53 | 27.03.12 | 1 | 680.8 | 19.2 | 113.7 | 12.4 | 2.6 | 306.2 | 3.1 | 380.6 | 128.4 | 155.7 | 547.9 | 0.3 |
|  |  | 10.04.12 | 5 | 556.3 | 16.8 | 79.4 | 16.2 | 1.9 | 403.6 | 2.1 | 376.7 | 73.2 | 223.9 | 549.6 | 0.2 |
|  |  | 24.04.12 | 9 | 521.2 | 15.5 | 71.6 | 16.0 | 1.5 | 400.8 | 2.3 | 353.5 | 71.4 | 223.4 | 527.5 | 0.1 |
|  |  | 8.05.12 | 13 | 577.6 | 17.0 | 85.0 | 16.0 | 1.6 | 401.6 | 2.1 | 381.6 | 85.0 | 224.3 | 560.9 | 0.2 |
|  |  |  |  |  |  |  |  |  |  |  |  |  |  |  |  |
| 2 | HK 56 | 27.03.12 | 1 | 664.4 | 18.8 | 107.6 | 16.1 | 1.6 | 398.6 | 1.5 | 430.1 | 113.4 | 225.0 | 612.9 | 0.2 |
|  |  | 10.04.12 | 5 | 858.7 | 29.9 | 146.3 | 17.1 | 4.1 | 345.5 | 0.9 | 601.6 | 191.7 | 165.5 | 717.7 | 0.5 |
|  |  | 24.04.12 | 9 | 834.6 | 20.8 | 133.3 | 18.1 | 3.1 | 466.0 | 0.4 | 528.4 | 136.1 | 263.0 | 571.8 | 0.1 |
|  |  | 8.05.12 | 13 | 638.6 | 18.6 | 111.5 | 13.5 | 3.6 | 287.9 | 1.3 | 468.4 | 153.0 | 161.1 | 561.6 | 0.4 |
|  |  |  |  |  |  |  |  |  |  |  |  |  |  |  |  |
| 3 | HK 60 | 27.03.12 | 1 | 517.3 | 13.0 | 87.6 | 8.2 | 2.2 | 172.0 | 3.2 | 232.1 | 102.0 | 79.0 | 332.9 | 0.2 |
|  |  | 10.04.12 | 5 | 569.1 | 15.9 | 100.4 | 9.9 | 1.5 | 156.0 | 1.4 | 291.6 | 123.4 | 74.9 | 173.7 | 0.0 |
|  |  | 24.04.12 | 9 | 635.5 | 14.6 | 117.9 | 10.1 | 2.4 | 163.5 | 1.8 | 320.9 | 148.1 | 81.7 | 354.1 | 0.2 |
|  |  | 10.05.12 | 13 | 818.8 | 22.5 | 131.4 | 19.1 | 2.8 | 257.8 | 0.5 | 453.6 | 151.7 | 92.7 | 309.6 | 0.1 |
|  | Mean |  |  | 656.1 |  |  |  |  |  |  |  |  |  |  |  |

| **Participant code** | | **Manganese (mg)** | **Zinc (mg)** | **Sulphur (mg)** | **Carotene (mcg)** | **Thiamine (mg)** | **Riboflavin (mg)** | **Niacin (mg)** | **Folic acid (mcg)** | **Vitamin C (mg)** | **Choline(mg)** | **Arginine (mg)** | **Histidine (mg)** | **Lysine (mg)** | **Tryptophan (mg)** |
| --- | --- | --- | --- | --- | --- | --- | --- | --- | --- | --- | --- | --- | --- | --- | --- |
| 1 | HK 53 | 0.9 | 1.6 | 244.0 | 187.0 | 0.4 | 0.5 | 3.9 | 57.0 | 7.8 | 46.8 | 1120.4 | 522.4 | 1094.5 | 196.5 |
|  |  | 0.4 | 0.7 | 318.9 | 232.7 | 0.3 | 0.6 | 2.0 | 51.2 | 9.8 | 19.2 | 718.8 | 412.2 | 1018.9 | 183.0 |
|  |  | 0.4 | 0.7 | 315.4 | 224.4 | 0.3 | 0.6 | 1.9 | 42.2 | 9.8 | 15.6 | 693.1 | 394.3 | 984.6 | 180.1 |
|  |  | 0.5 | 0.9 | 323.8 | 210.7 | 0.3 | 0.6 | 2.3 | 47.6 | 7.9 | 25.7 | 841.0 | 450.2 | 1085.2 | 197.3 |
|  |  |  |  |  |  |  |  |  |  |  |  |  |  |  |  |
| 2 | HK 56 | 0.7 | 1.3 | 327.7 | 236.4 | 0.4 | 0.6 | 3.7 | 52.8 | 9.8 | 27.5 | 1025.4 | 542.2 | 1199.4 | 215.1 |
|  |  | 1.4 | 2.9 | 302.8 | 311.5 | 0.6 | 0.6 | 5.7 | 122.1 | 7.8 | 95.6 | 2055.9 | 808.1 | 1752.4 | 337.5 |
|  |  | 1.0 | 2.1 | 329.9 | 230.4 | 0.5 | 0.7 | 5.4 | 47.6 | 12.8 | 0.0 | 1080.1 | 509.8 | 1047.2 | 238.0 |
|  |  | 1.7 | 2.0 | 291.7 | 198.6 | 0.5 | 0.5 | 4.7 | 50.7 | 7.8 | 12.8 | 937.3 | 479.8 | 928.3 | 218.1 |
|  |  |  |  |  |  |  |  |  |  |  |  |  |  |  |  |
| 3 | HK 60 | 0.6 | 1.1 | 127.7 | 130.4 | 0.3 | 0.3 | 3.0 | 34.4 | 5.8 | 27.5 | 718.2 | 343.8 | 659.3 | 114.0 |
|  |  | 0.9 | 1.5 | 111.1 | 125.6 | 0.3 | 0.3 | 4.0 | 24.8 | 6.4 | 0.0 | 1063.0 | 416.2 | 904.0 | 170.1 |
|  |  | 1.2 | 1.9 | 139.6 | 146.0 | 0.4 | 0.3 | 5.0 | 37.9 | 8.4 | 33.3 | 980.8 | 406.7 | 710.5 | 154.1 |
|  |  | 1.1 | 1.9 | 201.0 | 360.3 | 0.4 | 0.6 | 5.2 | 78.5 | 6.3 | 12.8 | 1501.8 | 594.6 | 1254.1 | 263.6 |

| **Participant code** | | **Phenylalanine (mg)** | **Tyrosine (mg)** | **Methionine (mg)** | **Cystine (mg)** | **Threonine (mg)** | **Palmitic acid (gm)** | **Stearic acid (gm)** | **Arachidic acid (gm)** | **Oleic acid (gm)** | **Linoleic acid (gm)** | **Linolenic acid (gm)** | **EPA** | **DHA** | **Total PUFA (gm)** |
| --- | --- | --- | --- | --- | --- | --- | --- | --- | --- | --- | --- | --- | --- | --- | --- |
| 1 | HK 53 | 968.9 | 667.2 | 405.7 | 192.1 | 706.7 | 0.4 | 0.1 | 0.1 | 1.4 | 0.9 | 0.0 | 0.0 | 0.0 | 0.9 |
|  |  | 770.3 | 615.7 | 350.3 | 136.6 | 610.2 | 0.4 | 0.1 | 0.1 | 1.4 | 0.9 | 0.0 | 0.0 | 0.0 | 0.9 |
|  |  | 737.4 | 606.4 | 346.0 | 132.3 | 596.0 | 0.4 | 0.1 | 0.1 | 1.4 | 0.9 | 0.0 | 0.0 | 0.0 | 0.9 |
|  |  | 839.3 | 661.3 | 381.6 | 155.8 | 663.6 | 0.4 | 0.1 | 0.1 | 1.4 | 0.9 | 0.0 | 0.0 | 0.0 | 0.9 |
|  |  |  |  |  |  |  |  |  |  |  |  |  |  |  |  |
| 2 | HK 56 | 1010.0 | 760.8 | 448.2 | 185.7 | 761.6 | 0.4 | 0.1 | 0.1 | 1.4 | 0.9 | 0.0 | 0.0 | 0.0 | 0.9 |
|  |  | 1536.4 | 1033.8 | 681.4 | 377.5 | 1180.9 | 1.1 | 0.3 | 0.2 | 2.8 | 1.6 | 0.0 | 0.0 | 0.0 | 1.6 |
|  |  | 981.0 | 825.2 | 512.0 | 240.8 | 794.8 | 0.5 | 0.1 | 0.2 | 1.9 | 1.2 | 0.0 | 0.0 | 0.0 | 1.2 |
|  |  | 948.2 | 682.1 | 390.3 | 281.4 | 681.6 | 0.5 | 0.1 | 0.2 | 1.9 | 1.2 | 0.0 | 0.0 | 0.0 | 1.2 |
|  |  |  |  |  |  |  |  |  |  |  |  |  |  |  |  |
| 3 | HK 60 | 637.5 | 415.2 | 256.2 | 121.7 | 437.7 | 0.4 | 0.1 | 0.1 | 1.4 | 0.9 | 0.0 | 0.0 | 0.0 | 0.9 |
|  |  | 734.6 | 634.8 | 441.8 | 194.3 | 650.1 | 1.4 | 0.4 | 0.2 | 4.0 | 2.4 | 0.1 | 0.0 | 0.0 | 2.5 |
|  |  | 766.3 | 556.2 | 354.6 | 184.4 | 564.3 | 0.6 | 0.1 | 0.2 | 2.4 | 1.5 | 0.0 | 0.0 | 0.0 | 1.4 |
|  |  | 1197.7 | 930.8 | 646.8 | 332.9 | 986.1 | 1.7 | 0.5 | 0.2 | 4.1 | 2.2 | 0.0 | 0.0 | 0.0 | 2.4 |
