# Supplementary material for: Effect of Native and Acetylated Dietary Resistant Starches on Intestinal Fermentative Capacity of Normal and Stunted Children in Southern India
Source: Int J Environ Res Public Health. 2019 Oct 15;16(20):3922. doi: 10.3390/ijerph16203922 (PMC6843365; doi:10.3390/ijerph16203922)
Supplement: Supplementary file 1 [file ijerph-16-03922-s001.zip › IJERPH_Rev1_Supp_materials/Table S4.docx]

| **S. No** | **Study Code** | **DOB** | **Gender** | **Height (cms)** | **Weight(kg)** |
| --- | --- | --- | --- | --- | --- |
| **HEALTHY CHILDREN** | | | | | |
| 1 | HK 02 | 2/09/2008 | M | 102 | 15.82 |
| 2 | HK 04 | 24/03/2009 | M | 94 | 15.9 |
| 3 | HK 16 | 19/07/2008 | M | 97 | 12.52 |
| 4 | HK 20 | 6/06/2009 | F | 93 | 13.85 |
| 5 | HK 21 | 20/05/2008 | F | 105 | 16.33 |
| 6 | HK 22 | 29/09/2009 | F | 90.5 | 12.01 |
| 7 | HK 38 | 21/03/2009 | M | 95 | 13.5 |
| 8 | HK 39 | 3/01/2009 | F | 99 | 12.55 |
| 9 | HK 40 | 15/04/2008 | F | 102.5 | 13 |
| 10 | HK 42 | 26/06/2007 | M | 106 | 16.63 |
| **Participants enrolled but subsequently excluded ('dropouts').** | | | | | |
| 11 | HK 41 | 27/06/2008 | F | 98 | 12.5 |
| 12 | HK 43 | 23/03/2010 | F | 83 | 9.68 |
| 13 | HK 44 | 24/03/2008 | M | 101 | 15.5 |
| 14 | HK 45 | 6/08/2009 | F | 90.3 | 11.8 |
| 15 | HK 46 | 5/03/2007 | M | 111 | 17.9 |
| 16 | HK 47 | 30/10/2008 | F | 98 | 13.93 |
|  |  |  |  |  |  |
| **STUNTED CHILDREN** | | | | | |
| 1 | HK 05 | 26.09.2009 | F | 81 | 8.76 |
| 2 | HK 11 | 27.05.2008 | F | 90 | 12.6 |
| 3 | HK 13 | 6.10.2007 | M | 97.5 | 12.78 |
| 4 | HK 49 | 30.01.2009 | F | 86.5 | 10.25 |
| 5 | HK 50 | 30.11.2009 | M | 82.5 | 10.09 |
| 6 | HK 51 | 5.11.2008 | M | 90 | 11.22 |
| 7 | HK 52 | 7.10.2009 | M | 84.9 | 10.72 |
| 8 | HK 53 | 26.09.2009 | M | 87 | 10.9 |
| 9 | HK 54 | 25.10.2007 | M | 97 | 13.85 |
| 10 | HK 55 | 3.10.2007 | F | 95 | 11.12 |
| 11 | HK 56 | 11.01.2008 | M | 92 | 12.27 |
| **Participants enrolled but subsequently excluded ('dropouts').** | | | | | |
| 12 | HK 57 | 27.07.2008 | M | 93 | 11 |
| 13 | HK 58 | 7.03.2008 | F | 90.5 | 10.22 |
| 14 | HK 59 | 28.10.2009 | F | 82.9 |  |
| 15 | HK 60 | 17.10.2007 | F | 93.5 | 12.6 |
| 16 | HK 61 | 26.02.2010 | F | 79 | 9.2 |
