# Supplementary material for: Effect of Native and Acetylated Dietary Resistant Starches on Intestinal Fermentative Capacity of Normal and Stunted Children in Southern India
Source: Int J Environ Res Public Health. 2019 Oct 15;16(20):3922. doi: 10.3390/ijerph16203922 (PMC6843365; doi:10.3390/ijerph16203922)
Supplement: Supplementary file 1 [file ijerph-16-03922-s001.zip › IJERPH_Rev1_Supp_materials/Table S5.docx]

|  | **Per 100 gm** |
| --- | --- |
| Moisture, g | 8.8 |
| Protein, g | 3.0 |
| Fat, g | 20.5 |
| Saturated fat, g | 12.8 |
| Total carbohydrate, g | 65.9 |
| Amylose, g | 20.6 |
| Sugar, g | 25.0 |
| Cholesterol, mg | 45 |
| Sodium, mg | 222 |
